# Editorial: COVID-19 and psychological disorders: From molecular basis to social impacts and therapeutic interventions

**DOI:** 10.3389/fpubh.2022.1000614

**Published:** 2022-08-19

**Authors:** Ghorbangol Ashabi, Elham Ahmadian, Elnara Shafiyeva

**Affiliations:** ^1^Department of Physiology, School of Medicine, Tehran University of Medical Sciences, Tehran, Iran; ^2^Kidney Research Center, Tabriz University of Medical Sciences, Tabriz, Iran; ^3^Department of Psychology, Social Sciences and Psychology Faculty, Baku State University, Baku, Azerbaijan

**Keywords:** COVID-19, mental health, health workers, PTSD, anxiety

Coronavirus disease 2019 (COVID-19) started in December 2019 in China and now is affecting 228 countries and territories (1). Mass quarantine, restriction of social activities, and stress on frontline health care workers have caused many psychological and mental health issues such as depression, cognitive and anxiety disorders to emerge (2, 3).

The pandemic side effects from the mental health crisis point of view have been focused on by many researchers worldwide. With this special Research Topic, we would like to explore the underlying probable molecular mechanisms affecting the brain's structure, chemistry, and functions in psychological disorders. We will also be discussing the social impacts of COVID-19 and how it has substantially affected people's lives. Including frontline health care workers who may require appropriate mental health support and treatment.

Regarding the COVID-19 pandemic on racial/ethnic minorities in U.S., Fisher et al. reported that Asian adults have more psychological issues such as distress in response to employment change, COVID victimization distress, and perceived increase in racial bias compared to Black and Latinx adults. Also, young adults were more vulnerable to depression and anxiety than older. Babicki et al. showed that women, those with lower education levels and residents of towns and cities exhibit a higher degree of both anxiety and depression symptoms.

In dementia-related diseases like Alzheimer's disease, Gan et al. investigated that confinement could increase rapid cognitive decline and ease cognitive deterioration during COVID-19 pandemic.

Moreover, studies on health care staff in the severe epidemic and low-risk areas by Lu et al., and Zhang et al., demonstrated that the COVID-19 pandemic increases the rate of post-traumatic stress syndrome (PTSD) and insomnia. Lu et al. showed that doctors, nurses, and other medical staff on the frontline of health care in Taiwan had a higher rate of PTSD, insomnia, and depression. Interestingly, Zhang et al. showed that PTSD and insomnia are seen even in non-medical staff in the low-risk epidemic area, which proposed that the COVID-19 pandemic could affect mental health problems in all healthcare workers individuals. Moreover, a mental health study on doctors and nurses by Jiang et al. stated that these medical healthcare workers faced significant complex multidimensional difficulties such as being worried about the impact on others, lack of knowledge, and being socially isolated. However, these doctors and nurses denied needing psychological support even when investigators informed them that it might be supportive. The results of a cross-sectional study in Bangladesh showed that frontline workers show higher rates of anxiety and depression compared to non-front-line workers. It was shown that organizational support and mental distress should be taken into account concomitantly in health care workers during the COVID-19 pandemic to escalate their resilience.

In non-health care workers such as teachers, anxiety, sleep disturbance and somatic symptoms have shown in increasing trend. On the other hand, it was reported that there is a high-risk of overall mental health complications, particularly a tighter connection of PTSD, in COVID-19 patients. Also, psychological distress was common in distinct type of patients such as organ transplant recipients during the COVID-19, and those with psychological symptoms had poorer quality of life or in chronic kidney diseases patients with stages 3 and 4 more anxiety symptoms were reported. Another important group were pregnant women. Expectant mothers should be given truthful and reliable information on the effect of COVID-19 on pregnancy. Job stability of individual during pandemic quarantine also significantly influenced the psychological symptoms. Physical and cognitive distresses mediate the association of attentional control and anxiety in COVID-19 patients.

An online questionnaire on 1,259 undergraduate university students by Ishimaru et al. verified that school over-adaptation group, older group, female subjects, shorter sleep time on weekdays, and belief that online education is not helpful had higher mental health problems during COVID-19 lockdown. Another study on internet-based mindfulness-based stress reduction (iMBSR) on breast cancer survivors by Kang et al. showed that iMBSR intervention reduced anxiety and depression, and insomnia during COVID-19 quarantine.

A lesson learned from COVID-19 pandemic demonstrates that it is important to provide supportive interventions to support the vulnerable groups by improving access to mental health services.

## Author contributions

GA, EA, and ES contributed to writing the topic editorial. All authors contributed to the article and approved the submitted version.

## Conflict of interest

The authors declare that the research was conducted in the absence of any commercial or financial relationships that could be construed as a potential conflict of interest.

## Publisher's note

All claims expressed in this article are solely those of the authors and do not necessarily represent those of their affiliated organizations, or those of the publisher, the editors and the reviewers. Any product that may be evaluated in this article, or claim that may be made by its manufacturer, is not guaranteed or endorsed by the publisher.
